# Clinical diagnostic exome evaluation for an infant with a lethal disorder: genetic diagnosis of TARP syndrome and expansion of the phenotype in a patient with a newly reported *RBM10* alteration

**DOI:** 10.1186/s12881-017-0426-3

**Published:** 2017-06-02

**Authors:** Zöe Powis, Alexa Hart, Sara Cherny, Igor Petrik, Erika Palmaer, Sha Tang, Carolyn Jones

**Affiliations:** 1 Ambry Genetics, 15 Argonaut, Aliso Viejo, CA 92656 USA; 2 Rush University Medical Center, Chicago, IL USA

**Keywords:** RBM10 protein, Human, Exome, Clinical diagnostic sequencing, TARP syndrome, Oligodactyly

## Abstract

**Background:**

Diagnostic Exome Sequencing (DES) has been shown to be an effective tool for diagnosis individuals with suspected genetic conditions.

**Case Presentation:**

We report a male infant born with multiple anomalies including bilateral dysplastic kidneys, cleft palate, bilateral talipes, and bilateral absence of thumbs and first toes. Prenatal testing including chromosome analysis and microarray did not identify a cause for the multiple congenital anomalies. Postnatal diagnostic exome studies (DES) were utilized to find a molecular diagnosis for the patient.

Exome sequencing of the proband, mother, and father showed a previously unreported maternally inherited RNA binding motif protein 10 (*RBM10*) c.1352_1353delAG (p.E451Vfs*66) alteration. Mutations in *RBM10* are associated with TARP syndrome, an X-linked recessive disorder originally described with cardinal features of talipes equinovarus, atrial septal defect, Robin sequence, and persistent left superior vena cava.

**Conclusion:**

DES established a molecular genetic diagnosis of TARP syndrome for a neonatal patient with a poor prognosis in whom traditional testing methods were uninformative and allowed for efficient diagnosis and future reproductive options for the parents. Other reported cases of TARP syndrome demonstrate significant variability in clinical phenotype. The reported features in this infant including multiple hemivertebrae, imperforate anus, aplasia of thumbs and first toes have not been reported in previous patients, thus expanding the clinical phenotype for this rare disorder.

**Electronic supplementary material:**

The online version of this article (doi:10.1186/s12881-017-0426-3) contains supplementary material, which is available to authorized users.

## Background

Since 2011, Diagnostic Exome Sequencing (DES) has proven to be cost effective and beneficial in providing a broad spectrum of previously undiagnosed patients with molecular genetic diagnoses while broadening the phenotype of known genetic diseases. The American College of Medical Genetics and Genomics recommends proper utilization of DES in the clinical assessment of individuals with a suspected genetic condition in which prior genetic testing fails to lead to a diagnosis [1]. The application of DES has allowed many undiagnosed patients who have endured an extensive diagnostic odyssey to receive a definitive genetic diagnosis [2–11].

Fetal and neonatal DES is being used more frequently in cases of infant demise, with or without congenital anomalies [4, 12]. Several studies have shown the clinical utility of DES for prenatal and neonatal patients due to increasing ease of DES and knowledge of the human genome [13–15].

Herein, we report a previously undiagnosed neonatal patient with a poor prognosis, who was identified to have a hemizygous *RBM10* alteration detected by DES establishing a molecular genetic diagnosis of TARP syndrome.

## Case presentation

### Clinical description

**Fig. 1:**
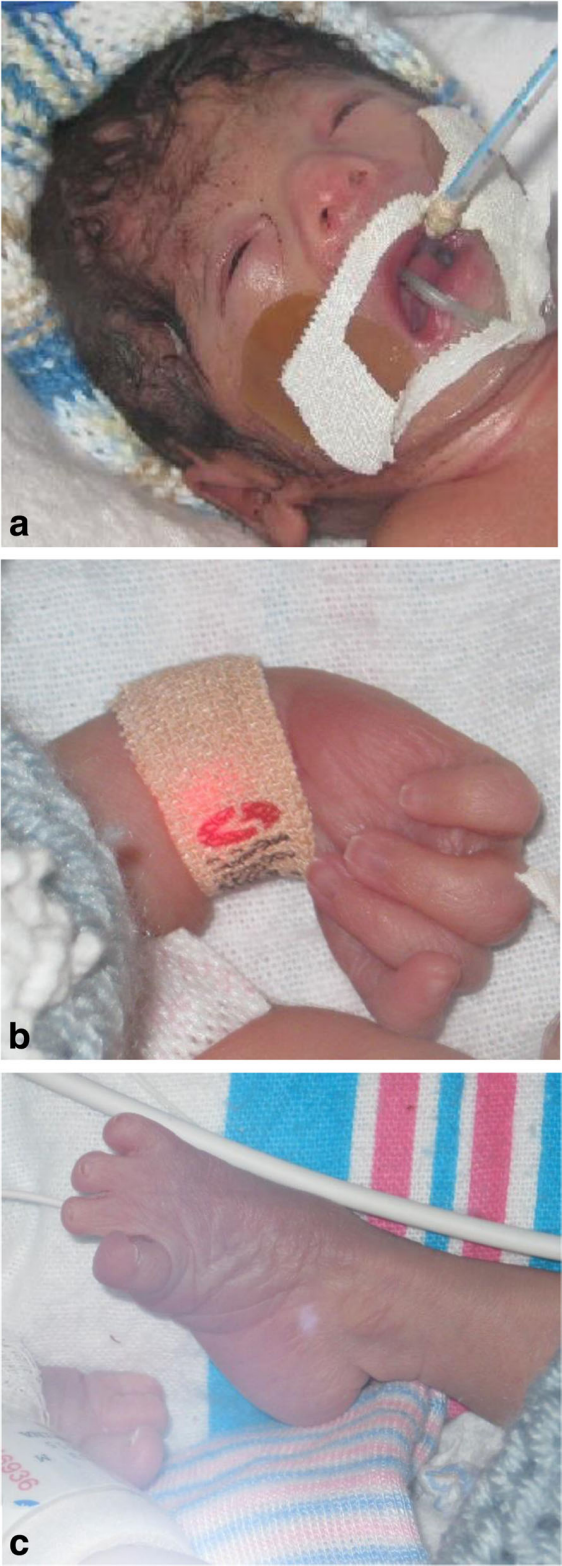
Clinical features. **a**. Features including brachycephaly, low set ears consistent with previous patients reported with TARP syndrome. **b**. Absent thumb. **c**. Aplasia of first toes

The male proband was born after a prenatal course complicated by anhydramnios and multiple fetal anomalies. Prenatal ultrasound identified bilateral renal aplasia, bilateral talipes, intrauterine growth restriction, and oligohydramnios that progressed to anhydramnios. Amniocentesis was performed during the pregnancy with normal karyotype and microarray. He was delivered at 35 weeks with a birth weight of 1.320 kg (<2%), length 40 cm (<3%) and head circumference 27 cm (<3%) to non-consanguineous parents of Mexican descent. Additional anomalies noted after birth included multiple hemivertebrae, imperforate anus, bilateral undescended testes, aplasia of thumbs and first toes, cleft palate, pulmonary hypoplasia, brachycephaly, and low-set ears (Fig. 1). A renal ultrasound demonstrated atrophic, dysplastic kidneys with scattered parenchymal cysts suggestive of cystic renal dysplasia. No other renal anomalies, such as hydronephrosis were seen. Multiple cardiac anomalies were identified at autopsy including atrial septal defect, unicuspid aortic valve, bicuspid pulmonic valve, and coarctation of the aorta. A head ultrasound showed an unusual head shape with flattening of the posterior calvarium, potentially related to brachycephaly with no other anomalies noted. An MRI was not performed. The patient's family history is unremarkable.

The infant’s prognosis was poor due to severe pulmonary hypoplasia. Whole exome testing was sent shortly after birth with a trio of the infant and parental samples. The infant died at day two of life from respiratory failure.

## Methods

Exome library preparation, sequencing, bioinformatics, and data analysis were performed as previously described [4, 16–18]. Briefly, samples were prepared using IDT xGen Exome Research Panel V1.0 and sequenced using paired-end, 150-cycle chemistry on the Illumina HiSeq 2500 (Illumina, San Diego, CA). Approximately 92% of characterized Mendelian disease genes are fully covered (100%) at > 20X (Farwell et al. [17]). The sequence data were aligned to the reference human genome (GRCh37) and variant calls were generated using CASAVA and Pindel [19]. Stepwise filtering included the removal of common SNPs, intergenic and 3’/5’ UTR variants, non-splice-related intronic variants, and lastly synonymous variants. Variants were then filtered further based on family history and possible inheritance models. Data are annotated with the Ambry Variant Analyzer tool (AVA) [20]. Identified candidate alterations were confirmed using automated fluorescence dideoxy sequencing.

## Results

Exome sequencing of the family trio (proband, mother, and father) resulted in a mean fold-coverage of captured regions of 135.04x per sample, with >98% covered with at least 10x coverage, and an average mean quality score of 34.56. (Additional file 1: Table S1). Family history inheritance model filtering based on autosomal and X-linked dominant and recessive and Y-linked inheritance models of the proband, mother, and father revealed 24 unique alterations in 17 candidate genes. Manual review of each alteration to rule out sequencing artifacts and polymorphisms along with medical interpretation to rule out genes lacking clinical overlap with the patient's evaluated phenotype (Additional file 2: Table S2) resulted in 1 gene (1 unique alteration) being considered as a candidate (Additional file 3: Table S3). A hemizygous alteration with potential clinical relevance, maternally inherited (*RBM10*) c.1352_1353delAG (p.E451Vfs*66) (Chr X:47040717-47040718), was confirmed by using automated fluorescence dideoxy sequencing (Additional file 4: Figure S1). To our knowledge, the c.1352_1353delAG alteration has not been previously reported in dbSNP, the NHLBI Exome Sequencing Project (ESP) [21], the ExAC database [22] or the 1000 Genomes database. [10].

**Fig. 2:**
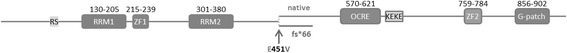
Domain structure diagram of RBM10 showing locations of domains (grey rectangles) and sequence biased regions (grey squares). Position of alteration shown with teal arrow, native sequence lost due to this alteration outlined in red, and the alternative 66 amino acid C-terminal sequence gained is depicted as a teal line. No structure or function was identified for this gained sequence

The full length protein spans 930 residues and consists of six identified domains (Fig. 2). This alteration results in loss of over half of the native protein sequence, including the octamer repeat (OCRE) [23], C2H2-Zinc, and G-patch domains. These domains have been shown, by truncation and domain deletion in the closely related protein RBM5, to be important for spliceosome assembly and recruitment to pre-mRNA [24] In particular, it has been shown that the OCRE domain binds to the C-terminal tail of snRNP core proteins [25]. Therefore, this alteration is predicted to cause loss of function either through nonsense-mediated decay or loss of functionally important protein domains and is interpreted as a pathogenic mutation.

## Discussion and conclusions

Whole exome sequencing and bioinformatics analysis of a male neonate of Mexican ancestry with multiple congenital anomalies was performed prior to his death at 2 days of age. Testing identified a maternally inherited hemizygous *RBM10* mutation. While rapid exome testing would not have benefitted this particular case, it is possible that rapid testing could aid additional patients with *RBM10* alterations in prognosis and knowledge to expand the clinical phenotype.

While previous testing had failed to provide a diagnosis for the proband, exome sequencing was able to determine a cause post-mortem based on the features evident before his death. This unique case highlights the power of whole exome sequencing as a diagnostic tool even after the patient is deceased. Post-mortem discovery of the cause of this patient’s disease may help the parents with future reproductive decision making. The family has the option of preimplantation genetic diagnosis, early prenatal diagnosis in a future pregnancy, adoption, or to avoid future pregnancies. In addition, carrier testing is available for at-risk maternal relatives including three maternal aunts to our proband. When an infant is born with multiple anomalies, identifying a cause is essential for the family to determine recurrence risk in a future pregnancy.

The *RBM10* gene is located on chromosome Xp11.23 and encodes the RNA binding motif protein 10 (OMIM 300080), originally called RNA binding protein S1-1, which participates in alternative splicing of apoptosis-related genes [26, 27]. Hemizygous alterations in *RBM10* cause the X-linked recessive disorder TARP (Talipes equinovarus, Atrial septal defect, Robin sequence, and Persistent left superior vena cava) syndrome. TARP syndrome is a disorder originally described by Gorlin et al. as lethal in males associated with talipes equinovarus, congenital heart defects, and Robin sequence (micrognathia, glossoptosis, and cleft palate) [28]. Additional findings reported in patients with pathogenic variants in *RBM10* include dysmorphic features (hypertelorism, ear abnormalities, wide mouth with downturned corners, upturned nose, supraorbital ridges), airway abnormalities, high arched palate, cryptorchidism, oligohydramnios, intrauterine growth restriction, failure to thrive, rocker bottom feet, hypotonia, cranial nervous system abnormalities, postaxial pes polydactyly, cutaneous syndactyly, sensorineural hearing loss, developmental delay, and seizures [26, 28, 29].

Our patient has the overlapping features of intrauterine growth restriction, complications of oligohydramnios, small size, cleft palate, pulmonary hypoplasia, a wide anterior fontanelle, cryptorchidism renal anomaly seen in other patients with TARP syndrome. His other features including multiple hemivertebrae, imperforate anus, aplasia of thumbs and first toes have not been reported in previous patients. While these additional features are present in other additional genetic conditions, due to the age of death, additional clinical diagnoses could not be made and a second pathogenic alteration was not found to explain these features.

The majority of cases reported have resulted in pre- or post-natal male lethality with the exception of two reported patients, one alive at 20 months and one alive at age 3 years and 7 months [26, 29]. Gripp et al. postulated that neonatal lethality was due to cardiac conduction defects, a finding not seen in our patient. To date, there have been no symptoms reported in carrier females and pathogenic variants reported in *RBM10* have been limited to loss of function alleles [26]. Therefore knowledge of the maternally inherited alteration may increase anxiety to due the recurrence risk, but can provide reassurance as additional health surveillance of the mother would not be necessary.

Previous reported cases of TARP syndrome demonstrate significant variability in clinical phenotype. Here, we report an additional patient with a pathogenic *RBM10* alteration demonstrating expansion of the clinical phenotype for this rare disorder. Diagnosis by DES is indispensable as the known phenotype of TARP expands and as limited time is often available to perform genetic testing in affected infants.

## Additional files

Additional file 1: Table S1. Run Metrics for family trio. Read depth values exclude the following: reads of low quality, reads that do not align uniquely to the exome, and PCR duplicates. (DOCX 22 kb)

Additional file 2: Table S2. Variant Filtering Based on Inheritance Model & Interpretation (DOCX 20 kb)

Additional file 3: Table S3. List of candidate genes/alterations resulting from inheritance model filtering and the manual removal of NGS artifacts and polymorphisms*. (DOCX 18 kb)

Additional file 4: Figure S1. Pedigree and Co-segregation. Familial pedigree and electropherograms of the c.1352_1353delAG (p.E451Vfs*66) alteration in the proband and additional family members. Shaded shapes indicate affected individuals. Asterisk (*) indicates whole exome sequencing performed. (DOCX 210 kb)

## Abbreviations

AVA: Ambry variant analyzer
dbSNP: Database of single nucleotide polymorphism
DES: Diagnostic exome sequencing
ESP: Exome sequencing project
OMIM: Online mendelian inheritance in man
*RBM10*: RNA binding motif protein 10
TARP syndrome: Talipes equinovarus, atrial septal defect, robin sequence, and persistent left superior vena cava syndrome

## References

[1] Directors ABo (2012). Points to consider in the clinical application of genomic sequencing. Genet Med.

[2] Bainbridge MN, Wang M, Burgess DL, Kovar C, Rodesch MJ, D'Ascenzo M, Kitzman J, Wu YQ, Newsham I, Richmond TA (2010). Whole exome capture in solution with 3 Gbp of data. Genome Biol.

[3] Beaulieu CL, Majewski J, Schwartzentruber J, Samuels ME, Fernandez BA, Bernier FP, Brudno M, Knoppers B, Marcadier J, Dyment D (2014). FORGE Canada Consortium: outcomes of a 2-year national rare-disease gene-discovery project. Am J Hum Genet.

[4] Farwell KD, Shahmirzadi L, El-Khechen D, Powis Z, Chao EC, Davis BT, Baxter RM, Zeng W, Mroske C, Parra MC (2014). Enhanced utility of family-centered diagnostic exome sequencing with inheritance model-based analysis: results from 500 unselected families with undiagnosed genetic conditions. Genet Med.

[5] Iglesias A, Anyane-Yeboa K, Wynn J, Wilson A, Truitt Cho M, Guzman E, Sisson R, Egan C, Chung WK (2014). The usefulness of whole-exome sequencing in routine clinical practice. Genet Med.

[6] Soden S, Saunders CJ, Willig LK, FArrow EG, Smith LD, Petrikin JE, LePichon JB, Miller NA, Thiffault I, Dinwiddle DL, Twist G, Noll A, Heese BA, Zellmer L, Atherton AM, Abelmoity AT, Safina N, Nyp SS, Zuccarelli B, Larson IA, Modrcin A, Herd S, Creed M, Ye Z, Yuan X, Brodsky RA, Kingsmore SF (2014). Effectiveness of exome and genome sequencing guided by acuity of illness for diagnosis of neurodevelopmental disorders. Sci Trans Med.

[7] Worthey EA, Mayer AN, Syverson GD, Helbling D, Bonacci BB, Decker B, Serpe JM, Dasu T, Tschannen MR, Veith RL (2011). Making a definitive diagnosis: successful clinical application of whole exome sequencing in a child with intractable inflammatory bowel disease. Genet Med.

[8] Yang JW, Han JY, Seong MW, Sung JJ, Park SS, Lee KW (2013). Hereditary Spastic Paraplegia with a Novel SPAST Mutation Misdiagnosed with Subacute Combined Degeneration. Experimental neurobiology.

[9] Rehm HL (2013). Disease-targeted sequencing: a cornerstone in the clinic. Nat Rev Genet.

[10] Bhattacharjee A, Sokolsky T, Wyman, S.K. Reese, M.G. Puffenberger E, Strauss K, Morton H. Parad R.B. Naylor, E.W. Development of DNA Confirmatory and High-Risk Diagnostic Testing for Newborns Using Targeted Next-Generation DNA Sequencing. Genetics in Medicine 2014(doi:10.1038/gim.2014.117):11. 10.1038/gim.2014.117 25255367

[11] Might M, Wilsey, M. The shifting model in clinical diagnostics: how next-generation sequencing and families are altering the way rare diseases are discovered, studied, and treated. Genet Med. 2014;doi:10.1038/gim.2014.23:2. 10.1038/gim.2014.23 24651604

[12] Yang Y, Muzny DM, Xia F, Niu Z, Person R, Ding Y, Ward P, Braxton A, Wang M, Buhay C (2014). Molecular findings among patients referred for clinical whole-exome sequencing. JAMA.

[13] Alamillo CL, Powis Z, Farwell K, Shahmirzadi L, Weltmer EC, Turocy J, Lowe T, Kobelka C, Chen E, Basel D (2015). Exome sequencing positively identified relevant alterations in more than half of cases with an indication of prenatal ultrasound anomalies. Prenat Diagn.

[14] Carss KJHS, Parthiban V, McMullan DJ, Maher ER, Kilby MD, Hurles ME (2014). Exome sequencing improves genetic diagnosis of structural fetal abnormalities revealed by ultrasound. Hum Mol Genet.

[15] Westerfield LE, Stover SR, Mathur VS, Nassef SA, Carter TG, Yang Y, Eng CM, Van den Veyver IB (2015). Reproductive genetic counseling challenges associated with diagnostic exome sequencing in a large academic private reproductive genetic counseling practice. Prenat Diagn.

[16] Farwell Gonzalez KD, Li X, Lu HM, Lu H, Pellegrino JE, Miller RT, Zeng W, Chao EC (2015). Diagnostic Exome Sequencing and Tailored Bioinformatics of the Parents of a Deceased Child with Cobalamin Deficiency Suggests Digenic Inheritance of the MTR and LMBRD1 Genes. JIMD Rep.

[17] Butterfield RJ, Stevenson TJ, Xing L, Newcomb TM, Nelson B, Zeng W, Li X, Lu HM, Lu H, Farwell Gonzalez KD (2014). Congenital lethal motor neuron disease with a novel defect in ribosome biogenesis. Neurology.

[18] Gandomi SK, Farwell Gonzalez KD, Parra M, Shahmirzadi L, Mancuso J, Pichurin P, Temme R, Dugan S, Zeng W, Tang S (2014). Diagnostic exome sequencing identifies two novel IQSEC2 mutations associated with X-linked intellectual disability with seizures: implications for genetic counseling and clinical diagnosis. J Genet Couns.

[19] Ye K, Schulz MH, Long Q, Apweiler R, Ning Z (2009). Pindel: a pattern growth approach to detect break points of large deletions and medium sized insertions from paired-end short reads. Bioinformatics.

[20] Laduca H, Stuenkel AJ, Dolinsky JS, Keiles S, Tandy S, Pesaran T, Chen E, Gau CL, Palmaer E, Shoaepour K (2014). Utilization of multigene panels in hereditary cancer predisposition testing: analysis of more than 2,000 patients. Genet Med.

[21] International HapMap C (2003). The International HapMap Project. Nature.

[22] (ExAC) EAC: ExAC Browser. In. http://exac.broadinstitute.org/; 2014.

[23] Callebaut I, Mornon JP (2005). OCRE: a novel domain made of imperfect, aromatic-rich octamer repeats. Bioinformatics.

[24] Bonnal S, Martinez C, Forch P, Bachi A, Wilm M, Valcarcel J (2008). RBM5/Luca-15/H37 regulates Fas alternative splice site pairing after exon definition. Mol Cell.

[25] Mourao A, Bonnal S, Soni K, Warner L, Bordonne R, Valcarcel J, Sattler M (2016). Structural basis for the recognition of spliceosomal SmN/B/B' proteins by the RBM5 OCRE domain in splicing regulation. Elife.

[26] Johnston JJ, Sapp JC, Curry C, Horton M, Leon E, Cusmano-Ozog K, Dobyns WB, Hudgins L, Zackai E, Biesecker LG (2014). Expansion of the TARP syndrome phenotype associated with de novo mutations and mosaicism. Am J Med Genet A.

[27] Inoue A, Yamamoto N, Kimura M, Nishio K, Yamane H, Nakajima K (2014). RBM10 regulates alternative splicing. FEBS Lett.

[28] Gorlin RJ, Cervenka J, Anderson RC, Sauk JJ, Bevis WD (1970). Robin's syndrome. A probably X-linked recessive subvariety exhibiting persistence of left superior vena cava and atrial septal defect. Am J Dis Child.

[29] Gripp KW, Hopkins E, Johnston JJ, Krause C, Dobyns WB, Biesecker LG (2011). Long-term survival in TARP syndrome and confirmation of RBM10 as the disease-causing gene. Am J Med Genet A.

