# Supplementary material for: Clinical diagnostic exome evaluation for an infant with a lethal disorder: genetic diagnosis of TARP syndrome and expansion of the phenotype in a patient with a newly reported RBM10 alteration
Source: BMC Med Genet. 2017 Jun 2;18:60. doi: 10.1186/s12881-017-0426-3 (PMC5455125; doi:10.1186/s12881-017-0426-3)
Supplement: Supplementary file 1 — Run Metrics for family trio. Read depth values exclude the following: reads of low quality, reads that do not align uniquely to the exome, and PCR duplicates. (DOCX 22 kb) [file 12881_2017_426_MOESM1_ESM.docx]

|  |  | |  |  | |  | |  |  |  | |
| --- | --- | --- | --- | --- | --- | --- | --- | --- | --- | --- | --- |
|  |  | |  | **Proband** | **Mother** | | **Father** | | **Average** | |  |
|  | **Sequence quality:** | | |  |  | |  | |  | |  |
|  |  | Mean Quality Score (PF*) | | 34.49 | 34.49 | | 34.71 | | **34.56** | |  |
|  | **Exome Read Depth:** | | |  |  | |  | |  | |  |
|  |  | Mean fold-coverage | | 131.76 | 131.76 | | 141.60 | | **135.04** | |  |
|  | **Base coverage spectrum:** | | |  |  | |  | |  | |  |
|  |  | %Base_10x (%) | | 0.98 | 0.98 | | 0.99 | | **0.98** | |  |
|  |  | %Base_20x (%) | | 0.97 | 0.97 | | 0.98 | | **0.97** | |  |
|  |  | %Base_50x (%) | | 0.88 | 0.88 | | 0.91 | | **0.89** | |  |

*Pass-filter
