# Supplementary material for: Clinical diagnostic exome evaluation for an infant with a lethal disorder: genetic diagnosis of TARP syndrome and expansion of the phenotype in a patient with a newly reported RBM10 alteration
Source: BMC Med Genet. 2017 Jun 2;18:60. doi: 10.1186/s12881-017-0426-3 (PMC5455125; doi:10.1186/s12881-017-0426-3)
Supplement: Supplementary file 2 — Variant Filtering Based on Inheritance Model & Interpretation (DOCX 20 kb) [file 12881_2017_426_MOESM2_ESM.docx]

|  | **Inheritance Model Filtering^1^** | **Manual Review^2^** | **Notable Candidate Genes^3^** |
| --- | --- | --- | --- |
| **Autosomal Dominant Genes (Alterations)** | 0 (0) | 0 (0) | **0 (0)** |
| **Autosomal Recessive Genes (Alterations)** | 5 (9) | 0 (0) | **0 (0)** |
| **X-linked Recessive Genes (Alterations)** | 2 (2) | 1(1) | **1(1)** |
| **X-linked Dominant Genes (Alterations)** | 0 (0) | 0 (0) | **0 (0)** |
| **Reduced Penetrance** | 12 (16) | 1(1) | **1(1)** |
| **TOTAL GENES (Alterations)** | **17 (24)** | **1(1)** | **1(1)** |

^1^Inheritance model filtering takes into account the co-segregation of variants along with disease presentation within the family trio. of allelic frequency (FAF) are applied at this stage; thresholds are <1% for recessive models of inheritance and <0.1% for dominant models of inheritance.

^2^The process of manual review involves the removal of genes unrelated to the patient's evaluated phenotype in addition to alterations that are considered benign. Novel genes are filtered out if the population frequency databases suggest they are functionally redundant.

^3^Notable Candidate Genes are those that are known to be associated with a disease phenotype that overlaps the proband's phenotype.

Note. During the 'Manual Review Process', characterized genetic etiologies that are unrelated to the patient's phenotype are excluded as candidates. At the same time, genes/ alterations are assessed alongside the patient's clinical presentation for their potential to constitute a novel genetic etiology. This assessment, involves the rigorous review of multiple lines of evidence to investigate and/ or establish a novel genetic etiology. Such evidence includes, but is not limited to animal models, functional/ expression experiments, protein interaction data, protein family conservation, and the frequency/ type of variation present in the population for the gene of interest. Further, there must be sufficient evidence to indicate that the candidate alteration(s) will impact the normal function of the gene product under consideration.
