# Supplementary material for: Clinical diagnostic exome evaluation for an infant with a lethal disorder: genetic diagnosis of TARP syndrome and expansion of the phenotype in a patient with a newly reported RBM10 alteration
Source: BMC Med Genet. 2017 Jun 2;18:60. doi: 10.1186/s12881-017-0426-3 (PMC5455125; doi:10.1186/s12881-017-0426-3)
Supplement: Supplementary file 3 — List of candidate genes/alterations resulting from inheritance model filtering and the manual removal of NGS artifacts and polymorphisms*. (DOCX 18 kb) [file 12881_2017_426_MOESM3_ESM.docx]

|  |  |  |  |  |  |  |  |  |  |  |  |  |
| --- | --- | --- | --- | --- | --- | --- | --- | --- | --- | --- | --- | --- |
| **Inheritance Model** | **Gene Symbol** | **Locus** | **RefSeq ID** | **Alteration** | **Genotype** | | | ***In silico* Prediction** | | **Allele Frequency (%)** | |  |
|  |  |  |  |  | **Proband** | **Father** | **Mother** | **PolyPhen** | **SIFT** | **ESP** | **1000 Genomes** |  |
| **Autosomal Dominant** | ***KCNQ1*** | **11:2592569** | **NM_000218** | **c.619G>A p.V207M** | **+/-** | **+/-** | **-/-** | **possibly damaging** | **deleterious** | **0.32%** | **1.7%** |  |
| **Autosomal Recessive** | **ATP7B** | **13:52508989** | **NM_000053** | **c.4301C>T p.T1434M** | **+/-** | **+/-** | **-/-** | **0.5%** | **0.94%** | **possibly damaging** | **tolerated** |  |
|  | **DACT1** | **14:59113464** | **NM_016651** | **c.2123C>G p.P708R** | **+/-** | **+/-** | **-/-** | **0.03%** | **N/A** | **benign** | **tolerated** |  |
|  | **DACT1** | **14:59113221** | **NM_016651** | **c.1880C>T p.A627V** | **+/-** | **-/-** | **+/-** | **N/A** | **N/A** | **benign** | **tolerated** |  |
|  | **MMP20** | **11:102465490** | **NM_004771** | **c.954-2A>T** | **+/-** | **-/-** | **+/-** | **0.29%** | **0.59%** | **N/A** | **N/A** |  |
|  | **NIPA1** | **15:23086365** | **NM_144599** | **c.45_47DELGGC p.A16DEL** | **+/+** | **+/-** | **+/-** | **N/A** | **N/A** | **N/A** | **N/A** |  |
| **X-Linked Recessive** | **RBM10** | **X:47040717** | **NM_005676** | **c.1352_1353DELAG p.E451VFS*66** | **+** | **-** | **+/-** | **N/A** | **N/A** | **N/A** | **N/A** |  |
|  | **ZNF81** | **X:47755310** | **NM_007137** | **c.248A>G p.E83G** | **+** | **-** | **+/-** | **benign** | **deleterious** | **N/A** | **N/A** |  |
| **Reduced Penetrance** | **BCHE** | **3:165504033** | **NM_000055** | **c.1584T>A p.Y528*** | **+/-** | **+/-** | **-/-** | **N/A** | **N/A** | **N/A** | **N/A** |  |
|  | **GP1BA** | **17:4837204** | **NM_000173** | **c.1305_1320DEL16 p.T436QFS*31** | **+/-** | **-/-** | **+/-** | **N/A** | **N/A** | **N/A** | **N/A** |  |
|  | **GP1BA** | **17:4837221** | **NM_000173** | **c.1322_1344DEL23 p.S441YFS*49** | **+/-** | **-/-** | **+/-** | **N/A** | **N/A** | **N/A** | **N/A** |  |
|  | **NOBOX** | **7:144101721** | **NM_001080413** | **c.138C>G p.Y46*** | **+/-** | **-/-** | **+/-** | **N/A** | **N/A** | **N/A** | **N/A** |  |
|  |  |  |  |  |  |  |  |  |  |  |  |  |
|  |  |  |  |  |  |  |  |  |  |  |  |  |
