# Supplementary figures and images for: Clinical diagnostic exome evaluation for an infant with a lethal disorder: genetic diagnosis of TARP syndrome and expansion of the phenotype in a patient with a newly reported RBM10 alteration
Source: BMC Med Genet. 2017 Jun 2;18:60. doi: 10.1186/s12881-017-0426-3 (PMC5455125; doi:10.1186/s12881-017-0426-3)

***5 YRS***

*

**P**

*

*


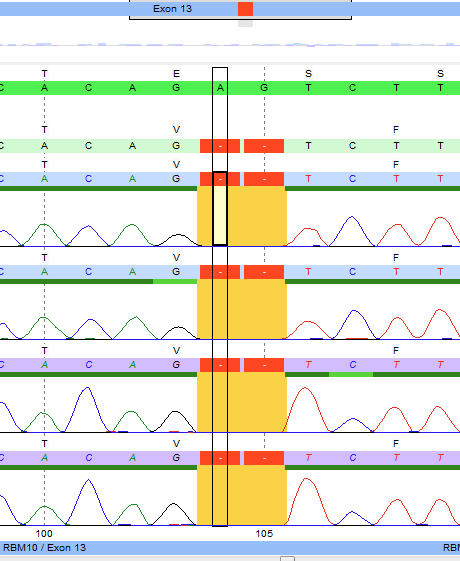

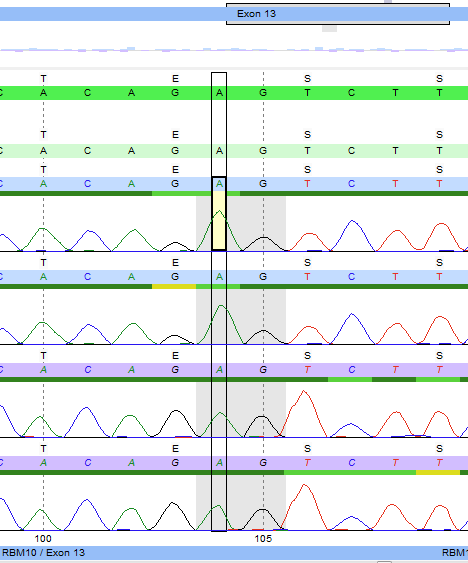

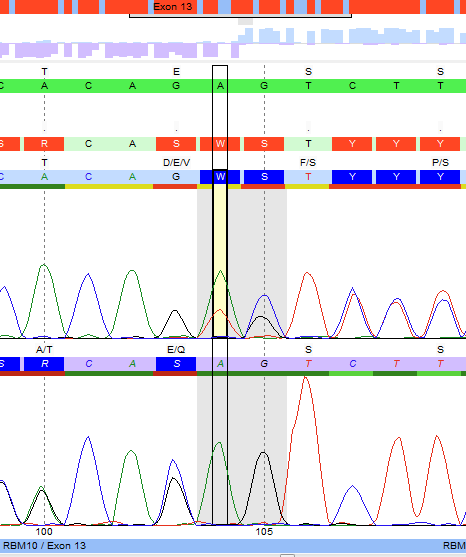

Supplement: Supplementary file 4 — Pedigree and Co-segregation. Familial pedigree and electropherograms of the c.1352_1353delAG (p.E451Vfs*66) alteration in the proband and additional family members. Shaded shapes indicate affected individuals. Asterisk (*) indicates whole exome sequencing performed. (DOCX 210 kb) [file 12881_2017_426_MOESM4_ESM.docx]
